# Application of nickel chitosan nanoconjugate as an antifungal agent for combating *Fusarium* rot of wheat

**DOI:** 10.1038/s41598-022-18670-2

**Published:** 2022-08-25

**Authors:** Divya Chouhan, Ankita Dutta, Anoop Kumar, Palash Mandal, Chandrani Choudhuri

**Affiliations:** 1 Nanobiology and Phytotherapy Laboratory, Department of Botany, University of North Bengal, Darjeeling, WB 734013 India; 2 ANMOL Laboratory, Department of Biotechnology, University of North Bengal, Darjeeling, WB 734013 India; 3 Department of Botany, North Bengal St. Xavier’s College, Jalpaiguri, WB 735134 India

**Keywords:** Plant sciences, Pathogenesis

## Abstract

Agro-researchers are endlessly trying to derive a potential biomolecule having antifungal properties in order to replace the application of synthetic fungicides on agricultural fields. Rot disease often caused by *Fusarium solani* made severe loss of wheat crops every year. Chitosan and its metallic nano-derivatives hold a broad-spectrum antifungal property. Our interdisciplinary study deals with the application of nickel chitosan nanoconjugate (NiCNC) against *Fusarium* rot of wheat, in comparison with chitosan nanoparticles (CNPs) and commercial fungicide Mancozeb. CNPs and NiCNC were characterized on the basis of UV–Vis spectrophotometry, HR-TEM, FESEM, EDXS and FT-IR. Both CNPs and NiCNC were found effective against the fungal growth, of which NiCNC at 0.04 mg/mL showed complete termination of *F. solani* grown in suitable medium. Ultrastructural analysis of *F. solani* conidia treated with NiCNC revealed pronounced damages and disruption of the membrane surface. Fluorescence microscopic study revealed generation of oxidative stress in the fungal system upon NiCNC exposure. Moreover, NiCNC showed reduction in rot disease incidence by 83.33% of wheat seedlings which was further confirmed through the observation of anatomical sections of the stem. NiCNC application helps the seedling to overcome the adverse effect of pathogen, which was evaluated through stress indices attributes.

## Introduction

It has been a great challenge over years for the agro-scientists to control fungal diseases that destroy a large scale of economically important food crops. Fungal pathogens made severe losses to the world agricultural production^1–4^. One such detrimental pathogen is *Fusarium* spp. that causes infection in a wide range of plant species. Majorly, it causes diseases like wilt, head blight, foot and root rot^5,6^. Foot and root rot of wheat (*Triticum aestivum* L.), occurring near the root-stem base, is one of the most common fungal diseases causing huge crop loss in major wheat growing areas of Europe, Asia, North America and Australia^7–10^. Several species of *Fusarium* are considered as phytopathogenic fungi against small grained cereals like wheat, barley, oats, etc.^11^. There are almost nine different species of *Fusarium* that causes rot disease in wheat as of reports published in 2020^12^. The species *Fusarium solani* is one of the most prevalent rot causing fungi in major economically important crop like wheat^13,14^. Foot and root rot of wheat is reported to be predominantly caused by *Fusarium solani* and *Fusarium oxysporum*^15,16^. Epidemics of *Fusarium* rot results in severe crop loss every year due to significant reduction in grain production and quality hindrance^17^. The rot disease attacks the basal portion of the plant and blocks the flow of water and nutrients to the foliage. Upon infection, several species of *Fusarium* produce health-threatening secondary metabolites called mycotoxins, which get accumulated in the plants and intake of which may be lethal to the human system^18^. The species *Fusarium solani* is the largest producer of T-2 toxin (T = Trichothecene) which is a precursor of neosolaniol, a neurotoxic compound^19^. Thus, the management of *Fusarium solani* rot in major economically important crops is crucial for minimizing the yield loss.

In recent years scientists have engineered versatile biomolecules such as nanoparticles (NPs) or nanoconjugates (NCs) and made their use to control fungal infections^20^. Several researchers have used chitosan for the synthesis of NPs or NCs due to its biocompatibility, greater permeability into biological membrane, cost-effectiveness, low toxicity and eco-friendly nature^21,22^. Scientists have already concluded chitosan as an antifungal agent owing to its polycationic nature that can bind to various negatively charged cellular components of fungal pathogens^23–25^. Conversion of chitosan into chitosan nanoparticles (CNPs) results in increasing its activity as a fungicidal component due to its increased surface area and greater encapsulation efficiency^26^. Next to CNPs, scientists have attempted for the synthesis of metallic conjugates of nanochitosan through versatile techniques, such as, Ionotropic Gelation Method, Emulsion Cross-linking, etc.^27,28^. But there are very few reports for the use of metallic nanochitosan as a promising antifungal agent. In comparison to chitosan and CNPs, metallic NPs conjugated with chitosan exhibit more biological activities due to its altered structural and functional properties such as, increased size and surface area, presence of more cationic group, active functional groups and greater condensing capacity^28^.

The control of *Fusarium* infection in major economically important crops is a very challenging task to the agro-researchers. Control strategies like crop rotation, use of botanicals, biological control and stubble management are in practice to the farmers but the success rate on controlling the rot disease was not satisfactory^29–32^. Synthetic chemical fungicides like Mancozeb through seed treatment or spraying are reported to control this disease to a greater extent^33^. Few reports from previous authors suggest the use of Mancozeb for imparting 50–100 percent inhibition of mycelial growth of *F. solani* responsible for causing foot and root rot disease^34,35^. But its excessive use is environmentally unfavorable due to its high toxic effect to the soil and crop system^36^. In this context, the consideration of an eco-friendly, biodegradable molecule like chitosan and its conjugates is more preferable. Anti-pathogenic activity of metallic nanoparticles like Ag, Cu, Zn, Fe, Ni, etc. has been reported previously by several workers^37^. Exposure of metal nanoparticles to the microbes generates oxidative stress that induces respiratory cells damage and increases protein leakage by disintegrating membrane permeability. A group of workers demonstrated the antifungal effect of silver nanochitosan against *Fusarium* species complex^38^. But the application of an expensive metal like silver for agricultural practices in developing countries is strenuous for the farmers as well as the government.

The metal Nickel is an essential micronutrient required especially for the growth and upliftment of chlorophyll content in wheat seedlings (1 mg kg^−1^ of fresh weight)^39^. Its deficiency in plants may negatively affect nitrogen metabolism, Fe uptake, plant growth and senescence. But higher level of Ni^2+^ exposure brings toxicity to the plants and gets accumulated in the soil giving rise to various health hazards^40^. There are also reports of Ni^2+^ ion for displaying cytotoxicity against a large number of phytopathogens by inducing disease resistance capacity in the plant system^37^. Nickel act as a constituent for several metalloenzymes, such as, urease, superoxide dismutase, methyl coenzyme M reductase, acetyl coenzyme A synthase, etc., as a result, its deficiency reduces the enzyme activity and affects nitrogen assimilation^41^. It is an inactive redox metal that does not generate Reactive Oxygen Species (ROS) directly. But high exposure level may lead to lipid peroxidation and electrolyte leakage in plants^42^. Thus, Nickel is a vital micronutrient essentially required for plant growth and proper functioning of their metabolism. Implementation of this vital metal at minimal concentration for the synthesis of nanoparticles will result in the enhanced bioactivity of the synthesized molecule against phytopathogens. Till now, very few information has been filed regarding the use of chitosan stabilized nickel nano particle against phytopathogens^43^. But no information was recorded yet regarding the bioactivity of Nickel Chitosan Nanoconjugate (NiCNC) against devastating pathogen *Fusarium*.

In our present study, we synthesized metallic nanoconjugate by incorporating a transitional metal and an essential micronutrient—nickel within nanochitosan meshwork by developing tri-polyphosphate cross-linking and applied the same for the control of *Fusarium* rot of wheat. The application of nickel nanochitosan conjugate for the management of *Fusarium* rot disease in wheat and improvement of seedling vigour through pathogenic stress tolerance capacity is a novel approach. The comparison between CNPs and NiCNC would help us to recognize the promotive effect of metallic nanoconjugates (NiCNC) in controlling *Fusarium* rot over non-conjugated chitosan nanoparticle (CNPs). In this context, Mancozeb was used for comparing the efficacy of synthesized nanochitosan and nickel-complexed nanochitosan with commercial synthetic chemical fungicides. Hence, data obtained from our study will assist to investigate the controlling measures for *Fusarium* rot in various other crops through biopolymer nanoconjugates.

## Results

### Synthesis and characterization of CNPs and NiCNC

**Figure 1:**
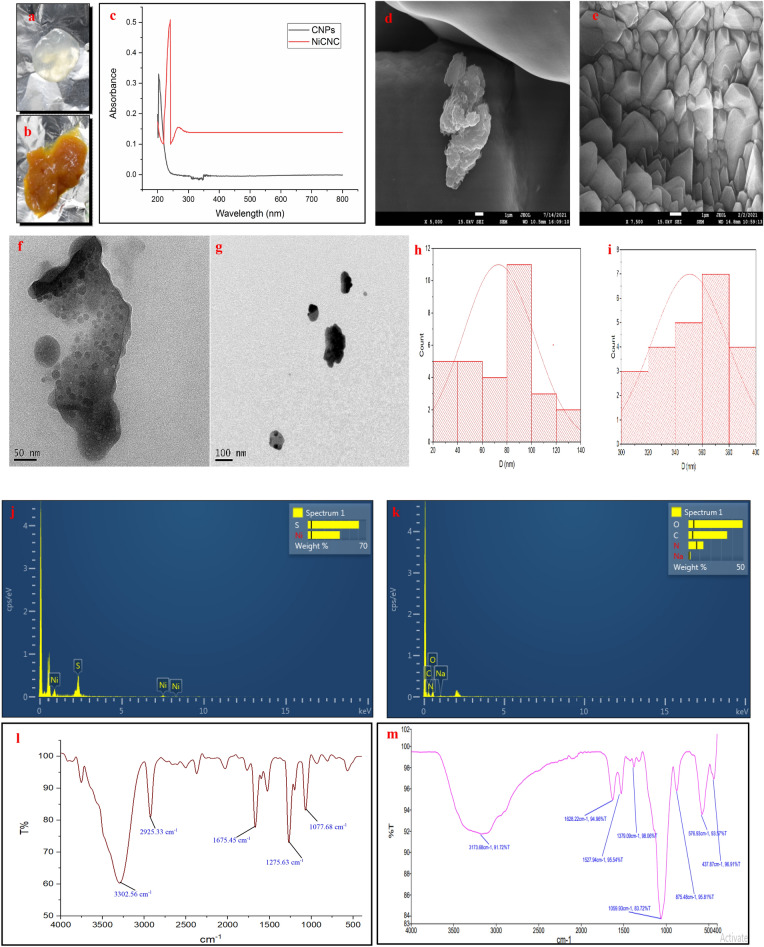
Nano-hydrogel images of (**a**) CNPs and (**b**) NiCNC obtained through Ionotropic Gelation Method; Characterization of CNPs and NiCNC through (**c**) UV–Vis spectra of CNPs and NiCNC; FE-SEM analysis of (**d**) CNPs and (**e**) NiCNC; HRTEM analysis of (**f**) CNPs and (**g**) NiCNC; Particle size distribution histogram of (**h**) CNPs and (**i**) NiCNC; EDXS analysis of (**j**) NiCNC and (**k**) CNPs and FT-IR analysis of (**l**) CNPs and (**m**) NiCNC.

As a primary observation, the formation of CNPs was indicated by the appearance of opaline solution and yellowish-orange for NiCNC hydrogel. The results for UV–visible absorption spectroscopy showed characteristics absorption peak for CNPs at 203 nm and NiCNC at 241 nm in the UV region. High Resolution Transmission Electron Microscopic (HR-TEM) analysis of CNPs showed fine spherical shaped nanoparticles of size ranging between 21 and 124 nm (measured through ImageJ Software), whereas, NiCNC showed particle size ranging between 300 and 400 nm. Both CNPs and NiCNC showed agglomerations with rough surface morphology entangled one upon another as revealed in Field Emission Scanning Electron Microscopic (FE-SEM) analysis. NiCNC showed hexagonal sheet like nano-disc morphology with pointed edges resembling high crystalline nature of the nanoparticle. Energy Dispersive X-ray Spectrometry (EDXS) spectrum analysis showed the presence of nickel in NiCNC which confirms it as a metal conjugated nanoparticle. All the necessary elements were confirmed in CNPs. Fourier Transformed Infrared Spectroscopy (FT-IR) spectral analysis showed almost varied peak positions of synthesized CNPs and NiCNC. FT-IR absorption peak at 3302.56 and 3173.68 cm^−1^ of CNPs and NiCNC, respectively, corresponds to O–H group and N–H group of stretching vibrations. The absorption band at 1675.45 and 1628.22 cm^−1^ showed the presence of amide I band in CNPs and NiCNC, respectively. The peak obtained at 1379.09 cm^−1^ signifies to the presence of –C–O–H in plane bending vibrations or –CH_2_ wagging or twisting in NiCNC. The absorption band at 1527.94 cm^−1^ resembles to N–H bending vibrations (amide II) in NiCNC which is absent in CNPs. The peak obtained at 1275.63 cm^−1^ for CNPs indicates the presence of P = O stretching vibrations. All the peaks obtained between 400 to 600 cm^−1^ in case of NiCNC are the characteristic peaks for metal oxide vibrations. Intense peak at 576.93 cm^−1^ obtained for NiCNC are due to the vibrations caused by Ni–O bond (Fig. 1).

### Effect of CNPs and NiCNC on mycelium radial growth

**Figure 2:**
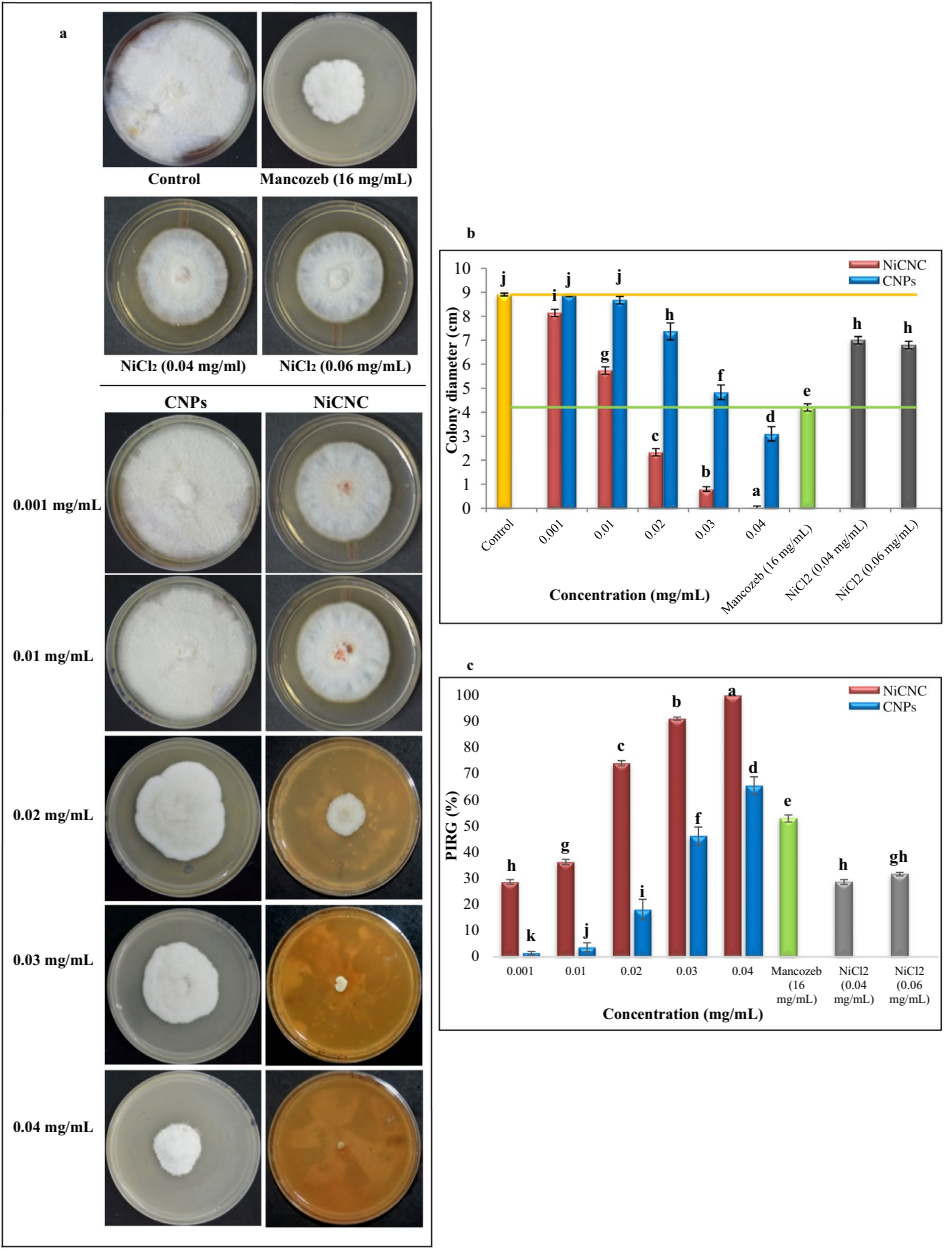
Effect of CNPs and NiCNC on mycelium radial growth of *F. solani*. (**a**) Mycelium radial growth of *F. solani* with different concentrations (0.001, 0.01, 0.02, 0.03 and 0.04 mg/mL) of CNPs and NiCNC on PDA plate; (**b**) Graphical representation of colony diameter of *F. solani* with different concentrations of CNPs and NiCNC in comparison with Mancozeb, NiCl_2_ and control at 7 days post-incubation; (**c**) Graph showing Percent Inhibition of Radial Growth (PIRG) of *F. solani* under tested concentrations of CNPs and NiCNC. Values are averages of three replicates (n = 3), error bars indicate standard deviation (SD) and different letters (a, b, c, etc.) indicate significant difference between treatments at p ≤ 0.05 by Duncan’s Multiple Range Test.

The antifungal activity of CNPs and NiCNC were evaluated by determining the colony diameter of *F. solani* grown in PDA media treated with different concentrations of both the nanoparticles. After 7 days of incubation, it was observed that in comparison to CNPs, NiCNC showed more efficient results with lesser colony diameter. Colony diameter reduced progressively with increasing concentration of CNPs and NiCNC. The commercial fungicide Mancozeb at its recommended dose showed insignificant activity with respect to NiCNC (p ≤ 0.05). The untreated plate showed full colony growth in 7 days (Fig. 2). 100% inhibition of radial growth was observed in case of NiCNC at 0.04 mg/mL, whereas, 0.04 mg/mL of CNPs showed 65.50% of growth inhibition. On the other hand, Mancozeb showed an inhibition percentage of 52.90 at its recommended dose. The antifungal activity of NiCl_2_ at 0.04 mg/mL and 0.06 mg/mL was found ineffective against the target fungus. Both the concentrations of NiCl_2_ showed 28% and 32% of mycelial growth inhibition.

### Effect of CNPs and NiCNC on inhibition of spore germination and sporulation of *F. solani*

**Figure 3:**
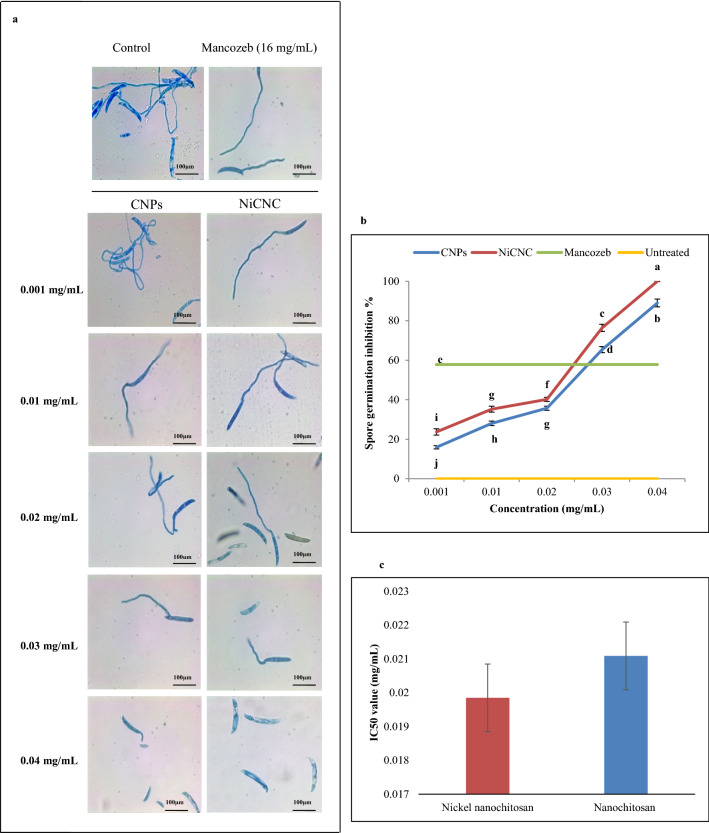
Effect of CNPs and NiCNC on inhibition percentage of spore germination of *F. solani*. (**a**) Microscopic images of spore germination inhibition of *F. solani* with different concentrations (0.001–0.04 mg/mL) of CNPs and NiCNC in comparison with Mancozeb (16 mg/mL) and untreated (control) spores (Magnification = 400×); (**b**) spore germination inhibition percentage of *F. solani*. Data are expressed as the mean ± SD (n = 3), different letters indicate significant difference between the treatments at p ≤ 0.05; (**c**) IC50 values of CNPs and NiCNC.

For further elucidation of antifungal activity of the nanoparticles, we investigated the spore germination inhibition percentage. When observed under light microscope after 6 h of treatment, a distinguished reduction in spore germination was observed in case of NiCNC treatment in comparison with untreated and CNPs treated spores. When the spores are exposed to 0.04 mg/mL of NiCNC solution, it showed 100% inhibition of spore germination, whereas, the dose of Mancozeb showed only 57.83% of inhibition of spore germination. On the other hand, 0.04 mg/mL of CNPs treatment on *F. solani* spores showed 89% inhibition of spore germination. The IC_50_ value of CNPs and NiCNC were obtained as 0.021 and 0.019 mg/mL, respectively (Fig. 3).

**Figure 4:**
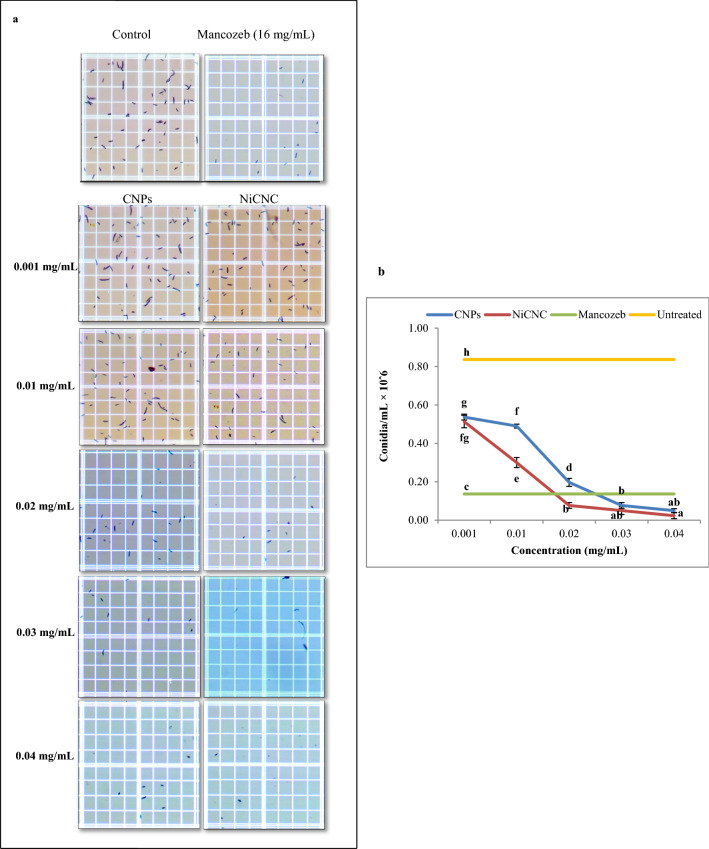
Effect of tested concentrations of CNPs and NiCNC on suppression of *F. solani* sporulation. (**a**) Sporulation count on heamocytometer observed under light microscope; (**b**) Graphical representation of the effect of CNPs and NiCNC in comparison with Mancozeb and untreated (control) spores on the formation of conidia; Values are averages of three replicates (n = 3), error bars indicate SD and different letters (a, b, c, etc.) indicate significant difference between treatments at p ≤ 0.05.

Effect of different concentrations of CNPs and NiCNC for 72 h on suppression of sporulation of *F. solani* revealed that treatment with NiCNC showed a significant decrease in the conidial formation with its increasing range of concentrations. The untreated plate showed 0.83 × 10^6^ conidia/mL, whereas, NiCNC and CNPs resulted in the formation of 0.02 × 10^6^ and 0.05 × 10^6^ conidia/mL, respectively at 0.04 mg/mL concentration. On contrary, the dose of Mancozeb produced 0.13 × 10^6^ conidia/mL (Fig. 4).

### Effect of CNPs and NiCNC on viability of spore and lipid peroxidation status of *F. solani*

**Figure 5:**
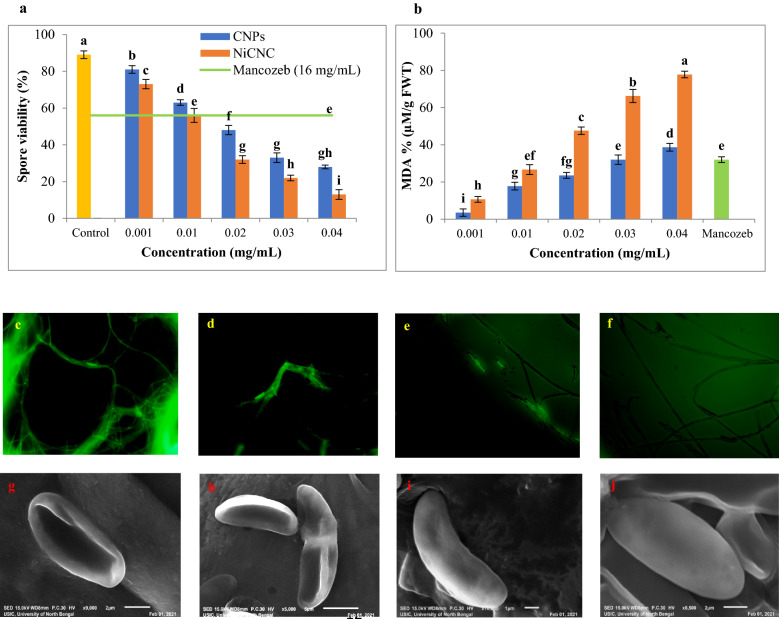
Effect of different concentrations of CNPs and NiCNC on (**a**) spore viability percentage and (**b**) MDA percentage of *F. solani*; Fluorescence microscopic observations showing generation of oxidative stress in *F. solani* conidia and mycelium due to the effect of (**c**) NiCNC (0.04 mg/mL), (**d**) CNPs (0.04 mg/mL), (**e**) Mancozeb (16 mg/mL) and (**f**) untreated mycelium; morphological changes of *F. solani* conidia directly exposed to (**g**) NiCNC (0.04 mg/mL), (**h**) CNPs (0.04 mg/mL), (**i**) Mancozeb (16 mg/mL) and (**j**) distilled water observed under scanning electron microscope.

Spores treated with NiCNC were found less viable with a viability percentage of 13 at 0.04 mg/mL, in comparison with untreated spores showing 90% viability. CNPs treatment (0.04 mg/mL) showed 28% of viable spores in the medium. Spores cultured with Mancozeb (16 mg/mL) showed 56% of viable spores (Fig. 5a).

The highest used concentration of NiCNC produces 77.77% of malonaldehyde (MDA) in the fungi, while, the untreated fungi having 0.00% of MDA. On the other hand, CNPs produces a maximum of 38.66% MDA when the fungi were treated with 0.04 mg/mL concentrations (Fig. 5b). In the contrary, the Mancozeb treatment showed 32.00% of MDA.

### Generation of ROS upon CNPs and NiCNC exposure on fungal system

The level of ROS generated in the fungal system after 72 h of nanoparticle application was measured by DCFH staining method. Figure 5c–f shows images observed under fluorescence microscope related to ROS level generated in untreated, NiCNC, CNPs and Mancozeb treated fungal mycelium. Our present study showed the generation of higher intensity of fluorescence in NiCNC treated fungal mycelium followed by CNPs and Mancozeb treatment showing moderate intensity of fluorescence. No expression of fluorescence was observed for untreated fungal mycelium.

### Ultrastructural changes of conidial membrane upon CNPs and NiCNC treatment

The application of synthesized nanoparticles for 72 h altered the morphology of the conidia of *F. solani.* Both the nanoparticles have induced pronounced depression in the conidial morphology resulting in complete distortion and severe damage of the membrane. The untreated fungal conidia showed a well-developed uniform surface morphology with no structural abnormalities. Mancozeb treatment resulted in slight depression in the conidial surface when compared with the untreated conidia; otherwise, there was no significant difference (Fig. 5g–j).

### Evaluation of disease incidence on wheat seedlings

**Figure 6:**
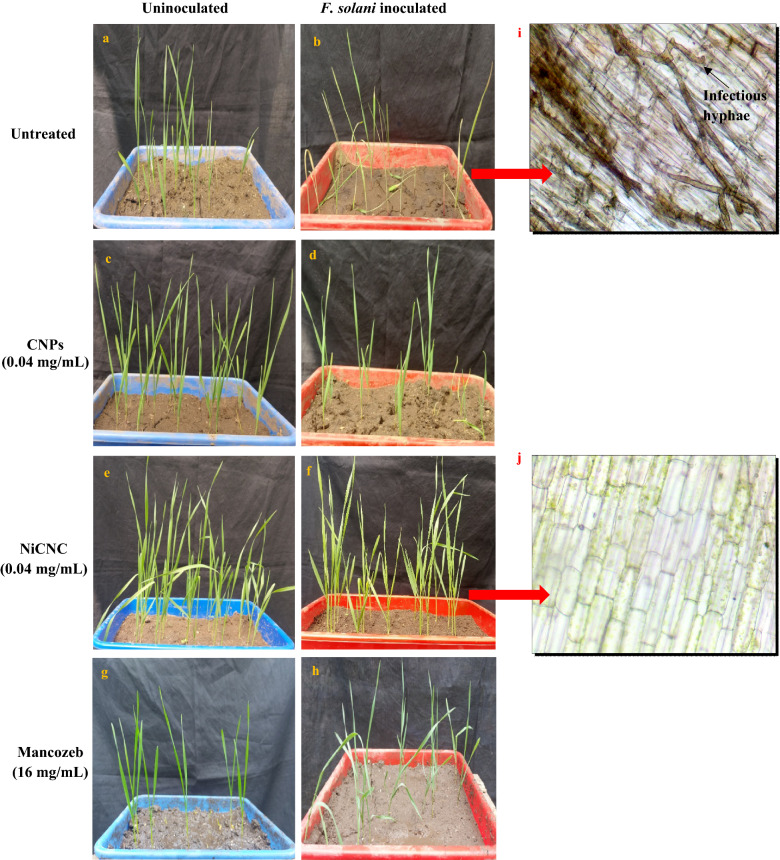
Disease symptoms of uninoculated and *F. solani* inoculated wheat seedlings at 20th day, exposed to (**a**,**b**) distilled water (untreated), (**c**,**d**) CNPs (0.04 mg/mL), (**e**,**f**) NiCNC (0.04 mg/mL) and (**g**,**h**) Mancozeb (16 mg/mL); (**i**) longitudinal section of stem of the distilled water treated and *F. solani* inoculated diseased seedling and (**j**) NiCNC treated and *F. solani* inoculated seedling. Infectious hyphae were observed to ramify only within the tissues of untreated seedlings.

At the end of 20th day, the symptoms on the inoculated seedlings were analysed based on 5-level scale of Santori and Infantino (2009) (Table 1). A significant reduction in the disease incidence was observed in NiCNC treated seedlings in comparison with untreated seedlings. The untreated seedlings showed 93.33% of disease incidence with dark brown coloration of the stem and root portion of the seedling. 36.66% dead plants were also noted on the tray after 20 days that could not survive after infection. But, NiCNC treated seedlings showed almost healthy vigour with only 16.67% of infection. Both, CNPs and Mancozeb treatment showed moderate amount of infection, 50% and 53.33%, respectively, on the seedlings (Fig. 6a–h).

**Table 1:** Representation for observation of seedling symptoms on the basis of 5-level scale by Santori and Infantino (2009), their disease index values.

5-level scale						Disease Index
Treatment	0	1	2	3	4	
	No. of seedlings					
Untreated	2	8	4	5	11	0.567^c^
CNPs	15	3	5	5	2	0.433^b^
NiCNC	22	5	3	0	0	0.267^a^
Mancozeb	14	6	5	3	2	0.467^b^

The values followed by the letter a, b and c showed significant difference at p ≤ 0.05.

For further confirmation of the incidence of disease in the seedlings, anatomical sections of the stem portion of both the untreated diseased and NiCNC treated inoculated soil grown seedlings were performed. Under microscope it was observed that the diseased seedlings showed brown coloured, chlorophyll less rotten cells with prominent occurrence of infection peg or appressorium that differentiate to form primary infectious hyphae which further developed into invasive hyphae. Moreover, these cells showed totally disrupted cell wall. In contrast to this, NiCNC treated seedlings showed fresh green and healthy cells with intact cell wall (Fig. 6i–j).

**Figure 7:**
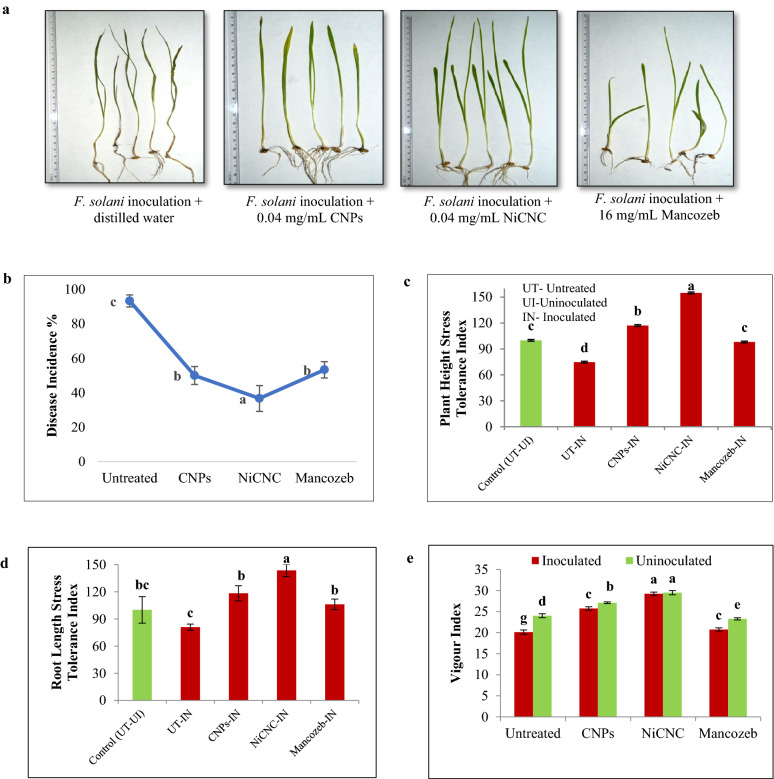
(**a**) Observation of morphological vigour of *F. solani* inoculated wheat seedlings at 20th day treated with distilled water, CNPs, NiCNC and Mancozeb; (**b**) disease incidence percentage of variably treated wheat seedlings; (**c**) Plant height and (**d**) Root length stress tolerance index of *F. solani* inoculated wheat seedlings treated with distilled water (untreated), CNPs, NiCNC and Mancozeb; (**e**) Vigour index of uninoculated and *F. solani* inoculated wheat seedlings at 20th day. Values are averages of three replicates (n = 3), error bars indicate SD and different letters (a, b, c, etc.) indicate significant difference between treatments at p ≤ 0.05.

Among all the treatments, seedlings with NiCNC treatment showed higher vigour index. Moderate vigour was observed in CNPs treated seedlings. Retarded vigour with short shoot and root length was observed in case of Mancozeb treated seedlings. Normal vigour pattern was observed in untreated seedlings (Fig. 7a,b). The effect of CNPs and NiCNC treatment in the mitigation of pathogenic stress on seedling vigour was evaluated through plant height and root length stress tolerance index. Seedlings inoculated with *F. solani* and further treated with 0.04 mg/mL of NiCNC showed maximum plant height and root length stress tolerance index. CNPs treated inoculated seedlings showed moderate stress tolerance index of plant height and root length. Inoculated seedlings treated with Mancozeb showed lower stress tolerance index than both the applied nanoparticles. Seedlings inoculated with *F. solani* without any treatment showed least tolerance of pathogenic stress among all the treatments (Fig. 7c–e).

### Cytotoxicity test of CNPs and NiCNC

Mammalian cell line were exposed to a range of increasing concentrations (0.01–0.4 mg/mL) of CNPs and NiCNC, to evaluate their cytotoxicity percentage at each concentration. Results clearly indicate the fact that at lower range of concentrations from 0.01–0.250 mg/mL, both the nanoparticles do not show any toxic effect to the seeded cells. The cells were not affected by the treatment of the nanoparticles and reproduced in a healthy manner. Therefore, cellular viability remained unchanged in presence of both the nanoparticles. But as the concentration of both the nanoparticles increases to 0.3 mg/mL, cells altered to give negative response and causes reproductive inability of kidney cells.

## Discussion

In relation to minimize the negative impact of synthetic fungicides on environment and public health concerns, the use of natural biological products like chitosan is gaining increased attention in the research world. Chitosan is regarded as a powerful chelating agent that can easily form complex with transition metals and heavy metals^44^. Various researchers have focused on the application of chitosan-metal complex in sequestration of metal ions, dyeing, water treatment, catalysis and various other industrial applications. However, a report suggests that chitosan-metal complex with bivalent ions (Cu, Zn, Ni, Fe) imparts promising anti-pathogenic activity than bulk chitosan or free metal salts due to the altered structural changes in chitosan molecule having -NH_2_ and -OH as dominating reactive sites^45^. Chitosan and nanochitosan is known to inhibit a broad spectrum of fungal pathogens, such as, *Alternaria solani*, *F. oxysporum*, *Aspergillus niger*, *Penicillium* spp., *Candida* spp., *Cordyceps militaris*, etc.^46–48^.

The use of Nickel chitosan nanoconjugate for combating *Fusarium* rot in wheat has resulted in significant reduction of disease incidence by 83.33%. Under in vitro examination, NiCNC at 0.04 mg/mL showed complete termination of the growth of fungal colony on PDA plate. It was observed that with the gradual increase in the concentration of NiCNC, the diameter of the fungal colony decreases. This confirms the dose dependent activity of metallic nanoparticles as suggested by different authors^49,50^. Our outcome suggests that NiCNC had more significant antifungal effect on *F. solani* over a dose of Mancozeb and tested concentrations of CNPs. Mancozeb reduced the fungal mycelial growth by 53%. It is a dithiocarbamate fungicide, classified by the Fungicide Resistance Action Committee (FRAC) having multi-site action^51^. Synthetic fungicides like, Mancozeb is reported to have numerous malfunctions in mammalian model organisms^52^. It acutely affects male fertility, gastrointestinal problems, scratchy throat, irritation, sneezing, coughing, bronchitis and transdermal absorption^53,54^. Information about potential toxicity of Mancozeb and other similar chemical fungicide, should not support its use in agricultural fields especially on globally consumed major food crops like wheat. It has been revoked in European Union since 2021 though used widely in different South-East Asian countries^55^. On the other hand, 0.04 mg/mL of CNPs showed 65.50% of growth inhibition which concludes it to be less efficient antifungal agent than NiCNC. The inductive effects of metallic nanoparticles or chitosan-metal complexes on different phytopathogens are already described to be more efficient by various researchers^56–59^. The antifungal activity of bulk NiCl_2_ solution at 0.04 mg/mL and 0.06 mg/mL was found incompetent against the *F. solani* growth. Even at a higher concentration of 0.06 mg/mL of NiCl_2_, the target pathogen is flourished with a remarkable colony growth. It is a clear indication that NiCl_2_ when punched with nanochitosan imparts potential antifungal activity at the aforesaid concentrations^60^. The application of Ag-chitosan nano-composite against *Fusarium* spp. showed full inhibitory action at 1 mg/mL whereas, in our study, NiCNC showed complete inhibition of *F. solani* at a very low dose (0.04 mg/mL)^28^. A thorough review of the previous work strongly supports the idea that Ni^2+^ ion on absorption into chitosan showed enhanced inhibitory effect against a number of plant pathogens^61^.

NiCNC is able to successfully inhibit the germination of *F. solani* spore at 0.04 mg/mL. Spores are the smallest propagative unit of any fungal pathogen that leads to the successful establishment of disease in the host plant^62^. Spore germination is very crucial for both vegetative and reproductive development of the pathogen. The germination of spore is very sensitive to abiotic stress, nutritional stress and host–pathogen communications^63^. The complete termination of spore germination process at highest tested concentration of NiCNC defines it as a potent sporicidal agent against *F. solani*. It was evident from the results obtained that NiCNC exhibited stronger antifungal activity than CNPs and Mancozeb as we found in case of radial colony growth.

Various researchers in their previous studies have recommended the use of metallic and metal oxide nanoparticles as antifungal agent^64^. Work done by Wani and Shah in 2012^65^ revealed that there was significant inhibition of spore germination in pathogens like *Fusarium oxysporum, Alternaria alternata* and *Rhizopus stolonifer* when their spores were treated with magnesium oxide and zinc oxide nanoparticles. More specifically, the absorption of Ni^2+^ ion into chitosan greatly affects the growth inhibition of *Candida albicans*^66^. NiCNC can evidently target the formation of conidia when exposed to highest tested concentration under suitable sporulation medium. An assumption may be drawn that the direct interaction between chitosan-metal complex and hyphae disrupts the cellular protein structure and its chemical nature, some of which are involved in conidiophore formation^67^. Furthermore, outcomes of our experiment are in agreement with the result obtained by Ahmed et al*.* (2016)^57^ where they elucidated that Ni- nanoparticle showed a significant raise in the sporicidal activity against *Fusarium* species at 50 ppm.

The colorimetric assay of XTT [2,3- Bis(2-methoxy-4-nitro-5-sulfophenyl)-5-[(phenyl-amino) carbonyl]-2H-tetrazolium hydroxide has been originally developed to verify the vegetative and reproductive viability of some clinically important fungi like *Candida* spp., *Saccharomyces cervisiae, Penicillium chryogenum, Aspergillus fumigatus, Aspergillus niger, Trichoderma atroviride*^68^. Sometimes the determination of minimum inhibitory concentration for fungi becomes difficult due to non-homogenous growth of hyphae and filaments. This colorimetric quantification of fungal growth is known to be more precise and accurate. This assay is known in use for bringing a complete inhibition in the metabolic activities within the fungal pathogens, as a result of which the spore germination and its viability gets affected^69,70^. It was evidently observed that with the increase in the dosage of NiCNC, the viability percentage of the spore decreases. Our results strongly support the outcomes obtained by Ghasemian et al. (2012) where they mentioned that metallic nanoparticles are effective in reducing spore viability percentage, particularly, for *Fusarium solani* and 60 mg/mL (minimum inhibitory concentration) of copper nanoparticles were found to be effective as a sporicidal agent, as demonstrated by them^68^. It is proved that the electrostatic interaction between the positive charges of the nanoconjugates and the negatively charged fungal cellular components leads to membrane destabilization and cellular protoplasmic leakage, thereof resulting in non-viable spores^38,71^.

The effect of NiCNC and CNPs on lipid peroxidation of fungi was evaluated by determining the MDA levels produced as a result of generation of intracellular stress due to the application of nanoparticles. The polyunsaturated fatty acids present in the fungal membrane contain methylene (–CH_2_–) group which are adversely affected by the generation of Reactive Oxygen Species (ROS) and thereby causes peroxidation of lipids. This phenomenon of lipid peroxidation completely disrupts the fungal membrane integrity and produces an aldehyde by-product MDA, an indicator of lipid peroxidation. This MDA produced will react with 2’-deoxyguanosine (M_1_G-dR) present in the fungal DNA to form a propane adduct. This process significantly influences the fungal metabolism by affecting various physiological functions of the cell including cell signalling, its growth and proliferation, differentiation and apoptosis^72^. In the present study, the MDA levels in fungi were found to be raised with the rising concentrations of NiCNC in comparison to control. The elevation of MDA levels is due to the deleterious effect of ROS generated as a result of its interaction with fungal membrane lipids. Our study demonstrated the maximum detrimental effect of NiCNC treatment on fungal cellular integrity in comparison to CNPs and Mancozeb.

The intensity of fluorescence observed in the fungal mycelia and conidia is directly proportional to the level of ROS generated due to stress on exposure to nanoparticles^73,74^. The fact that metallic nanoparticles can produce measurably more intracellular ROS than their bulk counterparts can be well concluded through our experiment^75^. It is observed in our study that 0.04 mg/mL of NiCNC produces maximum oxidative stress in fungal mycelium and conidia by the emission of high intensity of fluorescence, whereas, CNPs (0.04 mg/mL) and Mancozeb (16 mg/mL) produces moderate intensity of fluorescence. It can be clearly stated that NiCNC could generate maximum oxidative stress in the fungal system in comparison with CNPs and synthetic fungicide. ROS produced due to metallic nanoparticle treatment can completely hinder the membrane integrity and functionality of the fungi, thus leading to growth inhibition and death as well^72^. In a study, it was illustrated that the increment in the ROS level can imbalance the level of oxidant and antioxidants resulting in high oxidative stress followed by the release of cytochrome C that leads to cell apoptosis^76^. The agglomeration of metals into chitosan against *F. oxysporum* has been proven to generate high intensity of fluorescence that strongly supports our study^38^.

Both NiCNC and CNPs treatment on fungal conidia has resulted into severe irreversible damages on the conidial membrane including ruptured ends with increased membrane permeability and various surface anomalies analyzed through SEM. The application of NiCNC on the conidia results in losing its protoplasmic content followed by cellular leakage and seems to look like punctured conidia. This outcome was totally compatible with the results obtained by Dananjaya et al*.* (2017) on treatment of silver chitosan nanoparticle against *F. oxysporum* where they have observed cell wall damages and alteration in membrane permeability. It was confirmed earlier that metal oxide nanoparticles could be docked on the surface of the pathogenic bacteria^77^.

In vivo experiments revealed that 0.04 mg/mL of NiCNC provide maximum protection against *F. solani* attack in the developing wheat seedlings. This chitosan-metal conjugate was able to reduce the disease incidence to 16.67%. This concentration may be considered for the induction of resistance in wheat seedling against rot disease. Researchers have already established the biological role of chitosan and its nanoparticles as a resistance elicitor against fungal attack, more specifically against *Fusarium* spp.^78^. Various reports suggest that chitosan itself is effective in triggering plant innate immunity by showing low degree of symptom severity^79^. Scientists have affirmed that treatment with chitosan and its nano-derivatives could significantly enhance disease resistance among seedlings during germination stages^80^. Moreover, metallic conjugates of nanochitosan could further improve the disease resistance capacity as evaluated from disease incidence attribute of our study.

Not only for the prevention of rot disease but treatment with NiCNC can efficiently stimulate the seedling vigour index. Seedlings treated with NiCNC are taller with green healthy leaves having larger root length than the seedlings treated with synthetic fungicide, CNPs and distilled water. Seedlings treated with synthetic fungicides showed low vigour index due to retarded growth with short plant height and smaller roots that might depict the phytotoxic effect of the fungicide on the seedlings. This confirms the plant growth promoting activity of chitosan nanoparticles and its derivatives. Chitosan is known to be a plant growth regulator that enhances the biomass of the plant by decreasing the transpiration rate. In recent times, researchers have eager attention on the mechanism of plant growth promoting activity of chitosan-based nanoparticles^81,82^. It was proved that chitosan nanoparticles could significantly increase the seedling growth through enriched nutrient uptake that includes nitrogen, potassium, calcium, phosphorus and magnesium^83^. More precisely, it could be concluded that chitosan and its nano-derivatives promote the growth of germinating wheat seedlings through the activation of IAA signaling pathway^84,85^. Results obtained from our study are in agreement with the findings made by previous authors and it might be suggested that treatment with chitosan nanoparticles and its metallic derivatives could actively enhance the growth of the wheat seedlings under biotic stress exposure. Researchers have concluded that treatment with chitosan-based nanoparticles contributed in 1.5-fold increments of plant root and shoot length^86^. This increment was evident in our study, showing increased plant height after treatment with NiCNC when compared with untreated, CNPs and Mancozeb treated seedlings (Fig. 7e).

Ni^2+^ ion is also known to contribute in vegetative proliferation of the crops and deficiency of which may give rise to reduced vegetative growth, enhanced plant senescence, disturbed nitrogen metabolism, development of chlorosis, dwarfing of foliage, poor plant architecture and hindrance in normal Fe uptake^87^. Lower level of nickel exposure to the plants improves seed germination, chlorophyll synthesis and lateral root formation. Investigations revealed that nickel have a prominent role in the synthesis of phytoalexins and induction of disease resistance in plants^88^. As mentioned earlier in the introductory part, that Ni^2+^ ion serves as a co-factor of different metalloenzymes such as urease, carbon monoxide dehydrogenase, etc. Therefore, it is evident that nickel is an essential micronutrient required at lower concentration for normal functioning of the plant and amalgamation of nickel with chitosan may enhance its bioactivity when applied to plants. Literature reveals that crop plants requires an average of 3–1000 mg of Ni/kg of agricultural soil necessary for normal growth and functioning without any toxic effect^89^. In our experiment, the highest tested bioactive concentration i.e., 0.04 mg/mL of NiCNC hydrogel solution actually contains 38% of Ni (estimated through EDXS analysis), which stands an approximate of 0.00668 mg/mL of Ni applied to each seedling. Experiments were conducted in plastic trays containing 5 kg of soil for 30 seedlings. Each seedling was given treatment of 5 mL of NiCNC solution, therefore, for 30 seedlings 150 mL of NiCNC was applied in 5 kg of soil. Now, 150 mL of NiCNC would contain (0.00668 mg/mL × 150 mL) 1.002 mg/mL of Ni in 5 kg of soil (0.2 mg/mL Ni per kg soil) which is extremely low dose and have no chances of toxicity and metal accumulation in the soil^42^. Thus, the synthesized nickel chitosan nanoconjugate can be considered as eco-friendly and non-toxic antifungal agent^43^.

Stress tolerance analysis of the seedlings inoculated with *F. solani* revealed that application of NiCNC induces greater pathogenic stress tolerance capacity by accelerating the plant height and root length. It is known that stress caused due to pathogenic infection in plants may trigger plant height and affects root proliferation resulting in retarded growth. The seedlings treated with distilled water could not withstand pathogenic stress resulting in dead plants. Whereas, seedlings treated with CNPs showed moderate stress tolerance capacity in comparison with NiCNC treatment. Mancozeb treated seedlings resulted in lower tolerance of pathogenic stress next to CNPs treatment. Our study suggests that the activity of synthetic fungicide cannot successfully combat rot disease and invasion upon pathogen. Rather, excessive use of synthetic fungicides may lead to toxicity in plants, soil and environment^36^. Results obtained from this study clearly demonstrate the promotive effect of the metal (Ni^2+^) ion inserted in chitosan meshwork. The significance of insertion of nickel ion into chitosan network is to obtain the enhanced growth promoting activity of the wheat seedlings and uplifting of the anti-pathogenic activity over bulk chitosan.

Our experiments strongly suggest that NiCNC could actively eliminate the negative effect of pathogen in wheat seedling against the rot disease. Thus, the structural conformation of NiCNC becomes important regarding its antifungal activity. The formation of nanoparticles through Ionotropic Gelation Method is advantageous because it does not use any organic solvents and the processing conditions are mild which do not allow in bringing even a little change in the structure of the encapsulated drug^90^. UV–visible absorption spectroscopy showed characteristics absorption peak for NiCNC at 241 nm in the UV region, which closely resemble the formation of nanoparticles as described in previous reports^91^. Smaller particle size of the nanoparticle is advantageous due to its capability of easy penetration and higher cellular uptake capacity^92,93^. Particles of NiCNC showed size ranging between 300–400 nm. According to some previous reports, particle size below 500 nm is sufficient for easy penetration into fungal cell wall^94–97^. A group of workers already suggested that CNPs remain in the form of nano-aggregates, as indicated in Fig. 1f ^98^. Moreover, EDXS analysis of NiCNC showed the presence of Ni in chitosan network which confirmed the metallic nano-composite formation through electrostatic interactions between the hydroxyl groups of chitosan and the involving metal salt. This pattern of structure of NiCNC was already confirmed in previous studies made by various researchers^99^. FE-SEM analysis revealed agglomerations of both CNPs and NiCNC. This particular criterion of CNPs facilitates large surface area exposure is allowing it to be a material easy for adsorption^100^. Similarly, NiCNC showed uniformly distributed highly porous agglomerated nanoparticles. These agglomerations or formation of nano clusters and porous nature of NiCNC render them as useful biomolecules having excellent adsorption property and various other applications in the field of nano-medicine^101^. FT-IR analysis showed involvement of essential functional groups that perform active role in antifungal activity^102^.

**Figure 8:**
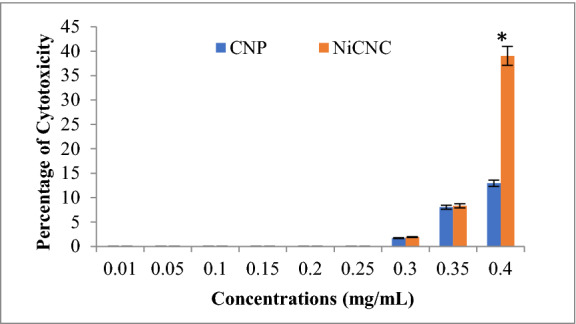
Comparison of cytotoxicity of CNPs and NiCNC on mammalian ACHN cells. Cellular toxicity was evaluated based on the percentage of cytotoxicity with different concentrations of CNPs and NiCNC (0.01–0.4 mg/mL). Data are expressed as the mean ± standard deviation (n = 3). Asterisk mark indicates significantly different mean values (p ≤ 0.05) between CNPs and NiCNC treatments.

Results for cytotoxicity assay do not show any significant difference with the increased concentrations of CNPs and NiCNC up to 0.25 mg/mL compared to untreated control (Fig. 8). Both the nanoparticles were found cytotoxic from 0.3 mg/mL and beyond. A significant difference in the percentage of cytotoxicity was noted at 0.4 mg/mL. The enhanced percentage of cytotoxicity noted for NiCNC is may be due to the involvement of metal ion in the nanoparticle^38^. The cytotoxicity analysis suggests that CNPs and NiCNC are non-toxic to ACHN cells up to 0.25 mg/mL whereas, the bioactive antifungal dosimetry applied to wheat seedlings against *F. solani* was 0.04 mg/mL which is much lesser than the toxic dose. Hence, the applied dose of NiCNC is non-toxic to human cells and can be referred to be used in agricultural fields for globally eaten major food crop like wheat.

**Figure 9:**
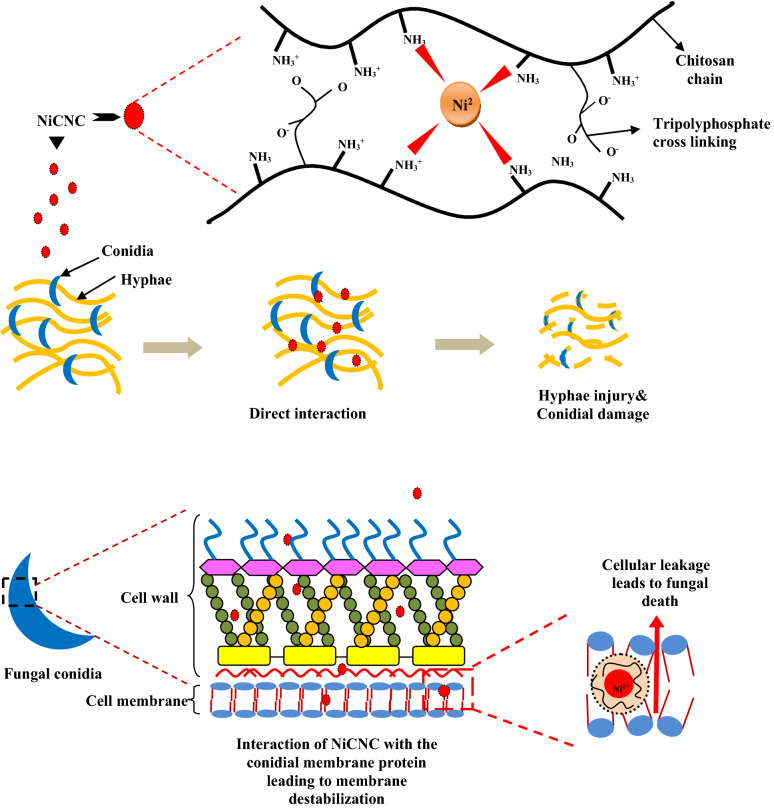
Schematic illustration for the mechanism of action of nickel chitosan nanoconjugate towards fungal pathogen.

Researchers have enlightened various concepts for describing the mode of action of chitosan and its derivatives in exhibiting antifungal activity. They claimed that the fungicidal activity could be due to the interplay between the positively charged chitosan molecules and negatively charged fungal cell surface components, which leads to increased membrane permeability following ionic imbalance^103^. It is also recommended that chitosan when enters into the fungal cell, interacts with the DNA by altering its structure thus inhibiting mRNA and protein synthesis^104^. Moreover, chitosan holds excellent metal binding capacity and is known to chelate the metal ions which are essential for microbial growth. The amine groups of polycationic chitosan are responsible for up-taking the metal ions^105^. The transitional metal ion nickel is assumed to form a centrally localized tetrahedral lattice structure with the positively charged amine groups of the chitosan^106^. This super positive metal core in the chitosan meshwork is responsible for the promotive antifungal activity by the dissolution of nickel ion that interrupt electron transport in the pathogenic cell leading to membrane destabilization, protoplasmic leakage followed by death of the fungi^107^ (Fig. 9).

There are various other management strategies used for controlling *Fusarium* rot infection in crop plants. Researchers have often used biocontrol agents like *Trichoderma* sp*.*, *Bacillus plumiles* and specific isolates of *Streptomyces* that reduces incidence of rot disease by 66–75%^108,109^. These biocontrol agents reduce but does not completely eradicate pest population neither promotes plant growth. Furthermore, the use of essential botanicals like extracts of *Azadirachta* sp*.*, *Zingiber* sp*.* and mustard-based botanicals showed higher efficacy in inhibiting *F. solani* mycelial growth^110^. But for field application in large scale, a promising formulation with stability and prolonged antifungal activity is necessary, especially against ascospores^111^. In this regard, the use of nickel chitosan nanoconjugate is advantageous because it facilitates greater disease reduction capacity with enhanced seedling health, performs complete eradication of the pathogen under in vitro examination and could retain prolonged antifungal activity. However, its wide scale field application is still required to be studied.

## Conclusion

CNPs and NiCNC were successfully synthesized by Ionotropic Gelation Method and characterized based on their various physio-chemical properties. The application of NiCNC exhibited potential growth inhibition property against *F. solani* and efficiently controlled the incidence of rot disease (under specific laboratory conditions). NiCNC treatment at 0.04 mg/mL completely terminates the mycelial colony growth, inhibits spore germination and conidia formation. NiCNC exposure to the fungal conidia produces ultra-structural damages that results into non-viable spores. Treatment with NiCNC produces high level of MDA in the pathogen resulting in increased lipid peroxidation of the fungi. The use of CNPs and synthetic fungicide Mancozeb cannot efficiently combat against rot disease. Our data recommends that NiCNC could be used as an antifungal agent in the management of rot disease caused by *F. solani*. NiCNC treatment generated high level of oxidative stress in the pathogen that ultimately terminated the ramification of the pathogen in the plant system. Also, application with NiCNC could potentially accelerate the seedling vigour index despite of biotic stress exposure. The concentration of nickel in the synthesized conjugate was minimal and proved to be non-toxic to the wheat seedling through cytotoxicity test and by promoting healthy seedling vigour. However, further studies are required to investigate the effect of NiCNC on wheat pathogens through field application.

## Materials and methods

### Preparation of CNPs and NiCNC

The preparation of CNPs was carried out by following Ionotropic Gelation Method^38^. Briefly, 0.2 g of low molecular weight chitosan (50,000–190,000 Da; 80% N-deacetylation; Product 448,869; Sigma Aldrich, USA) was dissolved in 1% (v/v) acetic acid solution and left under continuous stirring for 30 min. An anionic salt, Sodium Tripolyphosphate (STPP) (Na_5_P_3_O_10_; HSN Code: 28,353,100; SRL, India) at the concentration of 1 mg/mL (40 mL) was added drop-wise into the chitosan solution under constant magnetic stirring (REMI Equipments). A cloudy suspension formed spontaneously, resulted in NPs formation, was subjected to centrifugation at 10,000 rpm. The supernatant was discarded and pellet of CNPs gel was sun dried and stored at 4 $^\circ$C for further use.

NiCNC were prepared by following the method as described by Helen & Rani (2016) with minor modifications^43^. Its synthesis was carried out by adding drop-wise 4% aqueous solution of nickel chloride (NiCl_2_. 6H_2_O; RM760; Himedia, India) under constant magnetic stirring into chitosan solution. The solution was refluxed at 120 °C for 15 min. To this 0.05 M ascorbic acid (C_6_H_8_O_6_; MW 176.12; CMS1014; Himedia, India) was added followed by the addition of 2 mL of 0.6 M NaOH. After further stirring for 30 min, 0.5 mL phenyl hydrazine (C_6_H_6_N_2_Cl_2_.HCl; RM9448; Himedia, India) was added to the solution. The reaction was carried out further by the drop-wise addition of STPP under constant stirring followed by centrifugation at 10,000 rpm for 10 min. The supernatant was discarded, and the pellet was obtained as gel.

### Characterization of CNPs and NiCNC

The UV–Vis spectral absorbance of CNPs and NiCNC were collected by using UV–Vis Spectrophotometer (Aligent Technologies, Carry 100 UV–Vis) with scanning range of 200–800 nm of wavelength. The morphology of CNPs and NiCNC were examined by HR-TEM performed on a JEOL JEM 2100F with a magnification of 50x – 1.5 Mx and accelerating potential of 200 kV. The surface topography of the nanoparticles was examined by JSM-7900F Schottky Field Emission Scanning Electron Microscope (FE-SEM); JEOL, 0.1–30 kV. The presence of element down to boron, especially for NiCNC, was also determined by using EDXS. The functional groups present in the nanoparticles were detected through FT-IR analysis within the scanning range of 500–4000 cm^−1^ using KBr pellet technique. Before performing FT-IR analysis, the nanoparticles were sun-dried and grinded into fine powder using mortar-pestle. During analysis, 300 mg of dried KBr was crushed and mixed with 2 mg of previously dried nanoparticles and placed in grease free sample cup. A homogenous mixture of KBr and nanoparticle was prepared and used for sample spectral analysis.

### Collection, isolation and identification of pathogenic fungi

Diseased seedling having typical symptoms of stem and foot rot of wheat were collected from wheat growing fields of North Bengal, India for the isolation of target pathogen. Permissions were obtained from the farmers for collection of diseased plant parts. Diseased stem of about 1 cm were excised, surface sterilized and placed into sterile petri dishes containing 20 mL of autoclaved Potato Dextrose Agar (PDA) media. Plates are incubated for 7 days at 28 $^\circ$C. After 7 days the isolate was several times sub-cultured to obtain plates of pure culture. The pure culture was sent to IARI (Indian Agricultural Research Institute, New Delhi, India) for identification and the pathogen was reported to be *Fusarium solani* (I.D. No. 11116.19). The pure cultures were maintained in PDA slants at 28 $^\circ$C for further use in experiments^112^.

### Effect of CNPs and NiCNC on mycelium radial growth

The antifungal activities of CNPs and NiCNC on mycelium radial growth of *F. solani* were determined by poisoned food technique^38^. A range of concentrations (0.001, 0.01, 0.02, 0.03, 0.04 mg/mL) of CNPs and NiCNC were mixed with 20 mL of autoclaved Potato Dextrose Agar (PDA) media. A mycelium disc of 5 mm diameter was excised using sharp and sterile cork-borer from 7 days old sporulated culture of *F. solani* grown on PDA media and seeded at the center of each treatment plate. Each treatment plate was made upon three replicas. A plate treated with commercial fungicide, Mancozeb at its commercially recommended dose (16 mg/mL)^113^ and a plate without any treatment was prepared for comparison among all the treatments. The plates were incubated at 28 $^\circ$C for 7 days. Radial growth of the fungal colony was monitored daily until the fastest growing colony had covered the total diameter of the plate. The inhibition of the fungal growth was calculated using the following formula and expressed as percent inhibition of radial growth (PIRG)^38^:

$$\mathrm{PIRG }(\mathrm{\%}) =\frac{Radial\; growth\; of\; colony\; in\; control\; plate-Radial\; growth\; of\; colony\; in\; treated\; plate}{Radial\; growth\; of\; colony\; in \; control\; plate}\times 100$$

### Spore germination inhibition assay

The activity of CNPs and NiCNC on spore germination inhibition was assessed through concavity slide method. Conidial suspension was made by pouring 10 mL of sterile distilled water into the 7 days old sporulated culture tubes and gentle shaking followed by filtering the suspension with cheese cloth. Then, 150 µL of conidial suspension was mixed with equal volume of CNPs and NiCNC at different concentrations as used in the previous experiment. Similar set up was also prepared with 16 mg/mL of Mancozeb. About 60 µL of the hybrid containing an approximately of 1.0 × 10^6^ conidia/mL was put on the slide and incubated for 6 h at 28 $^\circ$C in a humid chamber. After 6 h, the conidia were examined under light microscope (Magnus, ch-20i, Olympus). The total number of germinated spores was counted out of the total spores present in six randomly chosen area of the slide under microscope. The spore germination inhibition % was calculated using the following formula^112^:

$$\mathrm{Spore\; Germination\; Inhibition}\mathbf{\%}=\frac{Germinated\; spores\; in\; control-Germinated\; spores\; in\; treatment}{Germinated\; spores\; in\; control}\times 100$$

### Sporulation suppression assay

The effect of CNPs and NiCNC on sporulation suppression was examined by using an appropriate sporulation inducing medium i.e., Czapek Dox Agar medium, mixed with CNPs and NiCNC solutions to obtain the final concentration as 0.001, 0.01, 0.02, 0.03, 0.04 mg/mL. The concentrations were compared with a blank and Mancozeb. After complete sterilization, 4 mL of the hybrid was poured in the Petri dish and allowed to cool. Then, fungal inoculum of about 0.5 cm diameter was transferred aseptically into the treated medium. The plates were incubated for 72 h at 28 $^\circ$C. The spore density for each treatment was measured using a haemocytometer under light microscope^71^.

### Spore viability assay

For the quantitative estimation of viable spores, a colorimetric assay was carried out using XTT 2,3- Bis(2-methoxy-4-nitro-5-sulfophenyl)-5-[(phenyl-amino)carbonyl]-2H-tetrazolium hydroxide (TC239, Himedia, India). This assay also involves an electron coupling agent Menadione (CAS No. 58-27-5, Sigma Aldrich, USA). Briefly, spore suspension (1.0 × 10^6^ conidia/mL) was prepared in Czapeck liquid medium and 100 µL of the suspension was cultured in 96 well flat bottom microplate for 4 h at 28 $^\circ$C. After incubation, 100 µL of both the nanoparticles at different range of concentrations (0.001, 0.01, 0.02, 0.03, 0.04 mg/mL) were added to the suspension and again incubated for another 4 h at same temperature. 50 µL of XTT solution at the concentration of 400 µg along with 7 µL of menadione (25 µM) was added to the suspension. The microplates were incubated for 3 h at 28 $^\circ$C and optical density was measured at 450 nm^114^.

### Determination of lipid peroxidation of *F. solani*

The effect of aforesaid concentrations of CNPs and NiCNC on lipid peroxidation of the fungi was determined by the quantitative estimation of malondialdehyde (MDA), an indicator of lipid peroxidation. Nanoparticles treated mycelial mat was harvested after 7 days of incubation at 28 $^\circ$C. The mycelial mat was washed thoroughly with sterile distilled water and then dried using blotting paper. 2 gm of the mycelia was cold homogenized with 10 mL of 0.1 M sodium phosphate buffer (pH 7.0) in a pre-chilled mortar and pestle. This homogenate was cold centrifuged at 10,000 rpm for 12 min. The cell free supernatant was used for MDA estimation. 100 µL of the homogenate was mixed with 3 mL of 0.335% (w/v) thiobarbituric acid (Himedia, India) solution containing 10% (w/v) trichloroacetic acid (Himedia, India). The mixture was subjected to boiling water bath for 15 min. The MDA level was determined in Spectrophotometer 169 (Systronics) and calculated with molar absorption co-efficient 1.56 × 10^5^ at 530 nm^115^.

### Analysis of ROS production upon CNPs and NiCNC treatment on *F. solani*

The oxidative stress assay was carried out to detect the level of ROS produced in fungal hyphae as an effect of CNPs and NiCNC treatment. A typical cell permeable, non-polar, non-fluorescent probe molecule 2’,7’-dichlorodihydrofluorescein diacetate (H_2_DCFH-DA) (D6665; Sigma Aldrich, USA) was used in this experiment. This hydrophobic molecule when enters inside the cell, gets deacetylated into 2’,7’-dichlorodihydrofluorescein (DCFH) by intracellular esterases. DCFH gets oxidized by ROS produced in the cell as a result of stress generation due to nanoparticle exposure. DCFH converts to Dichlorofluorescein (DCF) which gives detectable green fluorescence on excitation. The fungal hyphae were cultured in Potato Dextrose Broth (PDB) containing CNPs and NiCNC treatment at 0.04 mg/mL and 16 mg/mL Mancozeb for 72 h at 28 $^\circ$C. After 72 h the hyphae were collected by centrifugation at 10,000 rpm for 10 min. The media was discarded with supernatant. The hyphal cells were washed thrice with Phosphate Buffer Saline (PBS) and the hyphae were kept suspended in the same. The hyphal suspension was mixed with 40 µL of H_2_DCFH-DA in a dark chamber. The treatments were incubated for 2 h in the dark at 28 $^\circ$C with periodic shaking. Finally, the generation of ROS was observed visually under fluorescence microscope (Nikon Eclipse E200, Nikon, Tokyo, Japan) with excitation filter at 485 nm and emission filter at 525 nm^71^.

### Analysis of ultra-structural changes of conidial membrane upon CNPs and NiCNC treatment

The morphological changes in the fungal spore as a result of CNPs and NiCNC treatment at 0.04 mg/mL and Mancozeb at 16 mg/mL were observed under Scanning Electron Microscope (SEM) (JSM-IT 100; JEOL). The fungal spore in the PDB were treated with nanoparticles and incubated for 72 h. The fungal conidia were subjected to pre-treatment before microscopic examination^116^.

### Inoculation of plant material with pathogen and effect of CNPs and NiCNC treatment on rot disease

To evaluate the ability of CNPs and NiCNC in reducing the incidence of rot disease, a trial was performed. A Bread Wheat (*Triticum aestivum* L*.*) cultivar (PBW 343) collected from National Seed Corporation of India Ltd. was used for the experiment. All the plant experiments were in compliance with relevant institutional, national, and international guidelines and legislation. The isolated *F. solani* was used as inoculum for artificial inoculation in the seedlings to obtain symptoms of stem and foot rot in developing seedlings. The concentration of the inoculum was 10^6^ conidia/mL. Artificial inoculation of the pathogen was performed by root irradiation method. Briefly, 5 mL of conidial suspension was poured surrounding the stem base of 4 days old seedlings. After 24 h of inoculation, the same volume of 0.04 mg/mL of CNPs and NiCNC were applied following same procedure as above. Seedlings were grown in plastic trays (14 × 30 × 7 cm) containing 5 kg of soil mixed with vermiculite in 1:1 ratio. Treatments were categorized as follows: Untreated, CNPs (0.04 mg/mL) and NiCNC (0.04 mg/mL) and Mancozeb (16 mg/mL) treated seedlings. Each treatment was again divided into *F. solani* inoculated and uninoculated set. For each set 30 seedlings were sown in equal distance in the trays. The experiment was programmed for 20 days and regular watering was done. After the completion of 20 days, symptoms for rot caused by *F. solani* were recorded for each plant on the basis of a 5-level scale suggested by Santori and Infantino (2009)^117^: 0 = symptoms absent; 1 = slight dark brown coloration on the root or foot base; 2 = dark brown coloration on almost half of the stem or root; 3 = stem or root totally dark brown; 4 = death of the entire plant. The collected data were placed in McKinney formula (1923) for the evaluation of disease index^118^.

$$\mathrm{Disease\;index}=\frac{\sum_{k=1}^{k}{F}_{k}.{x}_{k}}{n{x}_{k}}$$

where F_k_ _=_ number of infected seedlings in the treatment plot (according to 5-level scale); n equals the total number of seedlings evaluated (n = 30); x_k_ equals the total number of infected seedlings from the control set up.

Disease incidence of the seedlings was also evaluated from the experiment^119^.

$$\mathrm{\% Disease\; Incidence}=\frac{Total\; no.\; of\; diseased\; plants}{Total\; no.\;of\; plants}\times 100$$

Morphological vigour of the treated seedlings was analyzed using the following vigour index formula^120^

$$\mathrm{Vigour\; index }=\left(Shoot\; length+Root \;length\right)\times Germination \%$$

### Stress tolerance analysis of the seedlings

Stress indices of the treated seedlings were calculated to evaluate the effect of CNPs and NiCNC treatment in the mitigation of pathogenic stress on seedling vigour^121^.

$$\mathrm{Plant\ Height\ Stress\ Tolerance\ Index\ }(\mathrm{PHSI}) =\frac{Height\ of\ the\ treated\ stressed\ plant}{ Height\ of\ the\ control\ plant}\times 100$$

$$\mathrm{Root\ Length\ Stress\ Tolerance\ Index\ }(\mathrm{RLSI}) =\frac{Root\ length\ of\ the\ treated\ stressed\ plant}{Root\ length\ of\ the\ control\ plant }\times 100$$

### Determination of cytotoxicity of CNPs and NiCNC

Human kidney cell line (ACHN) was procured from National Centre for Cell Science (NCCS), Pune, India. Cytotoxicity was examined by using MTT (3-(4,5-dimethylthiazol-2-yl)-2,5-diphenyltetrazolium bromide) assay^122,123^. Rapidly proliferating human kidney cell line was seeded in a 96 well micro titre plate at 37 °C, in presence of 5% CO_2_ in DMEM (Dulbecco’s Modified Eagle Medium) Ham F-12 cell culture medium at a density of 6 × 10^3^ cells/well. After 24 h, when the cells reached at a significant confluency, CNPs and NiCNC nanoparticles were added in each well at different concentrations ranging from 0.01- 0.4 mg/mL in triplicate. The treated plates were further incubated under same condition for 24 h. Next day, plates were withdrawn from incubator and cell culture media was aspirated. 10 µL of MTT dissolved in 1X PBS was added in each well and the plate was again kept for 3 h in the above-mentioned condition. Finally, 50 µl of Isopropanol, was added to each well containing MTT solution and was shaken for about 10 min. The absorbance was recorded at 620 nm in an ELISA reader.

The percentage of cell toxicity was calculated as [(C−T)/ C × 100], where “C” is the mean optical density of control (untreated cells) and “T” is the mean optical density of treated cells with different concentrations of nanoparticles.

### Statistical analysis

All the data obtained from the performed experiments were represented as the mean of six different observations with standard deviation (SD) (Mean ± SD). Statistical differences were analyzed by using Duncan’s Multiple Range Test (DMRT) at p ≤ 0.05 (DSAASTAT ver. 1.022), where the treatments that differ significantly were denoted by letter a, b, c, etc. Statistical analysis of the germination parameters between inoculated and uninoculated seedlings were performed using unpaired two-tailed *t*-test to find the significant difference between the different treatments using GraphPad Prism software ver. 6 for windows (GraphPad Software, Inc. USA) at p < 0.05.

## Supplementary Information

Supplementary Information 1.

Supplementary Information 2.

## Data Availability

All data generated or analyzed during this study are included in this published article and its supplementary information files.

## References

[1] Anderson PK, Cunningham AA, Patel NG, Morales FJ, Epstein PR, Daszak P (2004). Emerging infectious diseases of plants: Pathogen pollution, climate change and agrotechnology drivers. Trends Ecol. Evol..

[2] Brown JKM, Hovmoller MS (2002). Aerial dispersal of pathogens on the global and continental scales and its impact on plant disease. Science.

[3] Fisher MC, Henk DA, Briggs CJ, Brownstein JS, Madoff LC, McCraw SL, Gurr SJ (2012). Emerging fungal threats to animal, plant and ecosystem health. Nature.

[4] Flood J (2010). The importance of plant health to food security. Food Sec..

[5] Dweba CC (2017). *Fusarium* head blight of wheat: Pathogenesis and control strategies. Crop Protect..

[6] Morgavi DP, Wiseman J, Riley RT (2007). *Fusarium* and their toxins: Mycology, occurrence, toxicity, control and economic impact. Anim. Feed Sci. Technol..

[7] Burgess LW, Bryden WL (2012). *Fusarium*: A ubiquitous fungus of global significance. Microbiol. Aust..

[8] Abawi, G. S. & Pastor-Correales, M. A. Root rots of beans in Latin America and Africa: diagnosis, research methodologies, and management strategies. *Centro Internacional de Agricultura Tropical (CIAT), Cali*. **144**, (1990).

[9] Silva-Abud, L. E. B., Yoshida, F., Ulhoa, L., Wendland, A. & Lobo, J. M. Species identification and diversity of *Fusarium* spp. causing common bean root rot in Brazil. *Common Bean Disease Workshop on Angular Leaf Spot and Root Rot, Skukuza, South Africa.* 25 (2015).

[10] Balmas V, Scherm B, Marcello A, Beyer M, Hoffmann L, Migheli Q, Pasquali M (2015). *Fusarium* species and chemotypes associated with *Fusarium* head blight and *Fusarium* root rot on wheat in Sardinia. Plant Pathol..

[11] Liu Y, Wisniewski M, Kennedy JF, Jiang Y, Tang J, Liu J (2016). Chitosan and oligochitosan enhance ginger (*Zingiber officinale* Roscoe) resistance to rhizome rot caused by *Fusarium oxysporum* in storage. Carbohydr. Polym..

[12] Minati, M. H. First record of nine *Fusarium* spp. causing root rot in wheat (*Triticum aestivum* L.) in Iraq. *AIP Conference Proceedings* **2290**, 020009 (2020). 10.1063/5.0027398.

[13] Parikh L, Kodati S, Eskelson MJ, Adesemoye AO (2018). Identification and pathogenicity of Fusarium spp. in row crops in Nebraska. Crop Prot..

[14] Bahadur, A. Current Status of *Fusarium* and their management strategies. In: *Fusarium*—An Overview on Current Status of the Genus (2021). 10.5772/intechopen.100608.

[15] Ellis, M. L., Cruz Jimenez, D. R., Leandro, L. F. & Munkvold, G. P. Genotypic and phenotypic characterization of fungi in the *Fusarium oxysporum* species complex from soybean roots. *Phytopathol*. **104**, 1329–1339 (2014). 10.1094/PHYTO-02-14-0043-R 24983844

[16] Warren HL, Kommedahl T (1973). Prevalence and pathogenicity to corn of *Fusarium* species from corn roots, rhizosphere, residues, and soil. Phytopathol..

[17] Parry DW, Jenkinson P, Mcleod L (1995). *Fusarium* ear blight (scab) in small-grain cereals—A review. Plant Pathol..

[18] Drakopoulos D (2020). Control of *Fusarium graminearum* in wheat with mustard-based botanicals: from *in vitro* to in planta. Front. Microbiol..

[19] Ueno Y, Sawano M, Ishii K (1975). Production of Trichothecene Mycotoxins by *Fusarium* Species in Shake Culture. Appl. Microbiol..

[20] Banerjee A, Sarkar A, Acharya K, Chakraborty N (2021). Nanotechnology: an emerging hope in crop improvement. Lett. Appl. NanoBioSci..

[21] Hans ML, Lowman A (2002). Biodegradable nanoparticles for drug delivery and targeting. Curr. Opin. Solid State Mater. Sci..

[22] Sarkar, A. & Acharya, K. Chitosan: A promising candidate for sustainable agriculture in *Precision Agriculture and Sustainable Crop Production* (H. K. Chourasia, K. Acharya, V. K. Singh) 391–407 (India: Today and Tomorrow's Printers and Publishers, 2020).

[23] Malerba M, Cerana R (2016). Chitosan effects on plant systems. Int. J. Mol. Sci..

[24] Sathiyabama, M. R. & Charles, E. Fungal cell wall polymer-based nanoparticles in protection of tomato plants from wilt disease caused by *Fusarium oxysporum* f. sp. *lycopersici. Carbohydr*. *Polym*. **133**, 400–407 (2015). 10.1016/j.carbpol.2015.07.066 26344296

[25] Sathiyabama M, Parthasarathy R (2016). Biological preparation of chitosan nanoparticles and its *in vitro* antifungal efficacy against some phytopathogenic fungi. Carbohydr. Polym..

[26] Kong M, Chen XG, Xing K, Park HJ (2010). Antimicrobial properties of chitosan and mode of action: a state-of-the-art review. Int. J. Food Microbiol..

[27] Antonoglou O (2018). Nano-brass Cu-Zn nanoparticles as foliar spray non-phytotoxic fungicides. ACS Appl. Mater. Interfaces..

[28] Yanat, M. & Schroen. K. Preparation methods and applications of chitosan nanoparticles; with an outlook toward reinforcement of biodegradable packaging. *React. Funct. Polym.* **161**, 104849 (2021).

[29] Krupinsky JM, Bailey KL, McMullen MP, Gossen BD, Turkington TK (2002). Managing plant disease risk in diversified cropping systems. Agron. J..

[30] Asha, B. B., Chandra, N. S., Udaya, S. A. C., Srinivas, C. & Niranjana, S. R. Biological control of *Fusarium oxysporum f*. sp. *lycopersici* causing wilt of tomato with *Pseudomonas fluorescents*. *Int. J. Microbiol. Res*. **3**, 79–84 (2011).

[31] Cal A, Larena I, Sabuquillo P, Melgarejo P (2004). Biological control of tomato wilts. Res. Dev. Crop Sci..

[32] El-Rafai IM, Asswah SMW, Awdalla OA (2003). Biocontrol of some tomato diseases using some antagonistic microorganisms. Pakistan J. Biol. Sci..

[33] Getachew, Z. & Abeble, L. Effect of seed treatment using Mancozeb and Ridomil fungicides on *Rhizobium* strain performance, nodulation and yield of soybean (*Glycine max* L.). *J. Agric. Nat. Resour.* **4**(2), 86–97 (2021). 10.3126/janr.v4i2.33674.

[34] Bhaliya CM, Jadeja KB (2014). Efficacy of different fungicides against *Fusarium solani* causing coriander root rot. Bioscan.

[35] Padvi SA, Hingole DG, Khaire PB (2018). *In vitro* efficacy of fungicides against *Fusarium solani* incited by dry root rot of sweet orange. J. Pharmacogn. Phytochem..

[36] Roede, J. R. & Miller, G. W. Mancozeb in *Encyclopedia of Toxicology* (2^nd^ ed. Wexler, P.) 144–146 (Elsevier Inc., 2014).

[37] Chohan ZH, Pervez H, Khan KM, Rauf A, Maharvi GM, Supuran CT (2004). Antifungal cobalt (II), copper (II), nickel (II) and zinc (II) complexes of furanyl-thiophenyl-, pyrrolyl-, salicylyl- and pyridyl-derived cephalexins. J. Enzy. Inhib. Med. Chem..

[38] Dananjaya SHS, Erandani WKCU, Kim CH, Nikapitiya C, Lee J, Zoysa MD (2017). Comparative study on antifungal activities of chitosan nanoparticles and chitosan silver nano composites against *Fusarium oxysporum* species complex. Int. J. Biol. Macromol..

[39] Nielsen, F. H. Ultratrace Minerals in *Modern Nutrition in Health and Disease* (8th Ed. Shils M. E, Olson, J. A., Shike, M. & Ross, A. C.) 269–286 (Lea & Febiger, 1993).

[40] Gupta V, Jatav PK, Verma R, Kothari SL, Kachhwaha S (2017). Nickel accumulation and its effect on growth, physiological and biochemical parameters in millets and oats. Environ. Sci. Pollut. Res. Int..

[41] Ragsdale, S. W. Nickel Enzymes & Cofactors. *Ency. Inorg. and Bioinorg. Chem. (EIBC).* (2011). 10.1002/9781119951438.eibc0139.

[42] Genchi G, Carocci A, Lauria G, Sinicropi MS, Catalano A (2020). Nickel: Human health and environmental toxicology. Int. J. Environ. Res. Public. Health..

[43] Helen SM, Rani SM (2016). Synthesis, characterization and antibacterial activity of nickel chitosan nanoparticles. IOSR J. Appl. Chem..

[44] Adewuyi, S., Kareem, K. T., Atayese, A. O., Amolegbe, S. A. & Akinremi. C. A. Chitosan-cobalt (II) and nickel (II) chelates as antibacterial agents. *Int. J. Biol. Macromol*. **48**, 301–303. (2011). 10.1016/j.ijbiomac.2010.12.004 21145344

[45] Wang, X., Du, Y., Fan, L., Liu., H. & Hu, Y. Chitosan- metal complexes as antimicrobial agent: Synthesis, characterization and Structure-activity study. *Polym. Bull*. **55**, 105–113 (2005).

[46] Ziani K, Fernández-Pan I, Royo M, Mate JI (2009). Antifungal activity of films and solutions based on chitosan against typical seed fungi. Food Hydrocoll..

[47] Tikhonov VE (2006). Bactericidal and antifungal activities of a low molecular weight chitosan and its N-/2(3) -(dodec-2-enyl) succinoyl/-derivatives. Carbohydr. Polym..

[48] Guo Z (2006). Novel derivatives of chitosan and their antifungal activities *in vitro*. Carbohydr. Res..

[49] Tareq FK, Fayzunnesa M, Kabir S, Musrat N (2018). Evaluation of dose dependent antimicrobial activity of self-assembled chitosan, nano silver and chitosan-nano silver composite against several pathogens. Microb. Pathog..

[50] Phan TTV, Phan DT, Cao XT, Huynh TC, Oh J (2021). Roles of Chitosan in Green Synthesis of Metal Nanoparticles for Biomedical Applications. Nanomaterials.

[51] Yang LN (2019). Cross-resistance of pathogenic fungus *Alternaria alternata* to fungicides with different modes of action. BMC Microbiol..

[52] Gupta, P. K. Herbicides and fungicides in *Reproductive and Developmental Toxicology* (2nd ed. Gupta, R. C.) 503–521 (Academic press/ Elsevier, 2011).

[53] Robaire, B. & Hinton, B. T. The Epididymis in *Physiology of Reproduction* (4th Ed. Knobil & Neill) 691–752 (Elsevier Inc., 2015).

[54] Runkle J, Flocks J, Economos J, Dunlop AL (2017). A systematic review of Mancozeb as a reproductive and developmental hazard. Int. J. Environ. Res. Public Health..

[55] Commission Implementing Regulation (EU) 2020/2087. Concerning the non-renewal of the approval of the active substance mancozeb, in accordance with Regulation (EC) No 1107/2009 of the European Parliament and of the Council concerning the placing of plant protection products on the market, and amending the Annex to Commission Implementing Regulation (EU) No 540/2011. *Official Journal of the European Union*. (2020).

[56] Heinlaan M, Ivask A, Blinova I, Dubourguier HC, Kahru A (2008). Toxicity of nano-sized and bulk ZnO, CuO and TiO_2_ to bacteria *Vibrio fischeri* and crustaceans *Daphnia magna* and *Thamnocephalus platyurus*. Chemosphere.

[57] Ahmed IS, Yadav DR, Lee YS (2016). Applications of Nickel Nanoparticles for Control of *Fusarium* Wilt on Lettuce and Tomato. Int. J. Chem Tech Res..

[58] Ouda SM (2011). Antifungal activity of silver and copper nanoparticles on two plant pathogens, *Alternaria alternate* & *Botrytis cinerea*. Res. J. Microbiol..

[59] Wang X, Du Y, Fan L, Liu H, Hu Y (2005). Chitosan- metal complexes as antimicrobial agent: Synthesis, characterization and Structure-activity study. Polym. Bullet..

[60] Reddy V, Patil N, Angadi SD (2008). Synthesis, Characterization and Antimicrobial Activity of Cu(II), Co(II) and Ni(II) Complexes with O, N, and S Donor Ligands. Eur. J. Chem..

[61] Pivarciova L, Rosskopfova O, Galambos M, Rajec P (2014). Sorption of nickel on chitosan. J. Radioanal. Nucl. Chem..

[62] Judelson HS, Blanco FA (2005). The spores of *Phytophthora*: weapons of the plant destroyer. Nat. Rev. Microbiol..

[63] Li B, Lai T, Qin G, Tian S (2010). Ambient pH Stress Inhibits Spore Germination of *Penicillium expansum* by impairing protein synthesis and folding: A Proteomic-Based Study. J. Proteome Res..

[64] Chen JN (2016). Graphene oxide-silver nanocomposite: Novel agricultural antifungal agent against *Fusarium graminearum* for crop disease prevention. ACS Appl. Mater. Interfaces.

[65] Wani AH, Shah MA (2012). Unique and profound effect of MgO and ZnO nanoparticles on some plant pathogenic fungi. J. Appl. Pharm. Sci..

[66] Triawan, A., Pudyani, P. S., Marsetyawan, H. N. E., & Sismindari. S. The effect of nanochitosan hydrogel membrane on absorbtion of nickel, inhibition of *Streptococcus mutans* and *Candida albicans. Dent. J.* **48**(1), 26–36 (2015).

[67] Latijnhouwers M, Govers FA (2003). *Phytophthora infestans* G-protein β subunit is involved in sporangium formation. Eukaryot. Cell.

[68] Ghasemian E, Naghoni A, Tabaraie B, Tabaraie T (2012). *In vitro* susceptibility of filamentous fungi to copper nanoparticles assessed by rapid XTT colorimetry and agar dilution method. J. Mycol. Med..

[69] Clausen CA, Yang VW (2013). Colorimetric micro-assay for accelerated screening of mould inhibitors. Int. Biodeterior. Biodegradation..

[70] Khot PD, Suci PA, Tyler BJ (2008). *Candida albicans* viability after exposure to amphotericin B: assessment using metabolic assays and colony forming units. J. Microbiol. Methods..

[71] Chen J, Wu L, Lu M, Lu S, Li Z, Ding W (2020). Comparative Study on the fungicidal activity of metallic MgO nanoparticles and macroscale MgO against soil borne fungal phytopathogens. Front. Microbiol..

[72] Kalagatur, N. K., Ghosh., O. S. N., Sundararaj, N. & Mudili, V. Antifungal activity of chitosan nanoparticles encapsulated with *Cymbopogon martini* essential oil on plant pathogenic fungi *Fusarium graminearum*. *Front. Pharmacol.* **9**, 610 (2018). 10.3389/fphar.2018.00610 PMC5997812 29928233

[73] Kumar, K. N., Venkataramana, M., Allen, J. A., Chandranayaka, S., Murali, H. S. & Batra, H. V. Role of *Curcuma longa* L. essential oil in controlling the growth and zearalenone production of *Fusarium graminearum*. *LWT Food Sci. Technol.* **69**, 522–528 (2016).

[74] LeBel CP, Ischiropoulos H, Bondy SC (1992). Evaluation of the probe 2′, 7′-dichlorofluorescin as an indicator of reactive oxygen species formation and oxidative stress. Chem. Res. Toxicol..

[75] Li Y, Zhang W, Niu J, Chen Y (2012). Mechanism of photo generated reactive oxygen species and correlation with the antibacterial properties of engineered metal-oxide nanoparticles. ACS Nano.

[76] Xia Z, Lundgren B, Bergstrand A, De Pierre JW, Nassberger L (1999). Changes in the generation of reactive oxygen species and in mitochondrial membrane potential during apoptosis induced by the antidepressants imipramine, clomipramine, and citalopram and the effects on these changes by Bcl-2 and Bcl-X(L). Biochem. Pharmacol..

[77] Jiang W, Mashayekhi H, Xing B (2009). Bacterial toxicity comparison between nano- and micro-scaled oxide particles. Environ. Pollut..

[78] Ing, L. Y., Zin, N. M., Sarwar, A. & Katas, H. Antifungal activity of chitosan nanoparticles and correlation with their physical properties. *Int. J. Biomater.* Article ID 632698 (2012) 10.1155/2012/632698. 10.1155/2012/632698 PMC3399401 22829829

[79] Agrawal GK, Rakwal R, Tamogami S, Yonekura M, Kubo A, Saji H (2002). Chitosan activates defense/stress response(s) in the leaves of *Oryza sativa* seedlings. Plant Physiol. Biochem..

[80] Yin H, Zhao X, Guang YD (2010). Oligochitosan: A plant diseases vaccine—A review. Carbohydr. Polym..

[81] Kumaraswamy RV (2018). Engineered chitosan-based nanomaterials: Bioactivities, mechanisms and perspectives in plant protection and growth. Int. J. Biol. Macromol..

[82] Chouhan D, Mandal P (2021). Applications of chitosan and chitosan based metallic nanoparticles in Agrosciences -A review. Int. J. Biol. Macromol..

[83] Van SN, Minh HD, Anh DN (2013). Study on chitosan nanoparticles on biophysical characteristics and growth of Robusta coffee in green house. Biocatal. Agric. Biotechnol..

[84] Li, R. et al. Effects of chitosan nanoparticles on seed germination and seedling growth of wheat (*Triticum aestivum* L.). *Int. J. Biol. Macromol.* **126**, 91–100 (2019). 10.1016/j.ijbiomac.2018.12.118 30557637

[85] Khati, P., Chaudhary, P., Gangola, S., Bhatt, P. & Sharma, A. Nanochitosan supports growth of *Zea mays* and also maintains soil health following growth. *3 Biotech* **7**(1), 81 (2017). 10.1007/s13205-017-0668-y PMC5429309 28500403

[86] Zhao, T., Deng, X., Xiao, Q., Han, Y., Zhu, S. & Chen, J. IAA priming improves the germination and seedling growth in cotton (*Gossypium hirsutum* L.) via regulating the endogenous phytohormones and enhancing the sucrose metabolism. *Ind. Crops Prod.* **155**, 112788 (2020).

[87] Graham, R. D., Welch. R. M. & Walker, C. D. A role of nickel in the resistance of plants to rust. *Austr. Agron. Soc. Proc.* **159**, (1985).

[88] Wood, B. W. & Reilly, C. C. Interaction of nickel and plant disease in *Mineral Nutrition and Plant Disease* (ed. Datnoff, L. E., Elmer, W. H. & Huber, D. M.) 217–247 (MN: American Phytopathological Society Press, 2007).

[89] Liu, G., Simmone, E. H. & Li, Y. Nickel nutrition in plants, University of Florida. HS1191.

[90] Nagpal K, Singh SK, Mishra DN (2010). Chitosan nanoparticles: a promising system in novel drug delivery. Chem. Pharm. Bull..

[91] Triwulandari E, Fahmiati S, Sampora Y, Meliana Y, Ghozali Md, Sondari D (2013). Effects of some parameters on particle size distribution of chitosan nanoparticles prepared by ionic gelation method. J. Clus. Sci..

[92] Bhattarai N, Ramay HR, Chou SH, Zhang M (2006). Chitosan and lactic acid-grafted chitosan nanoparticles as carriers for prolonged drug delivery. Int. J. Nanomed..

[93] Labhasetwar V, Song C, Levy RL (1997). Nanoparticle drug delivery system for restenosis. Adv. Drug Deliv. Rev..

[94] Akter MJ, Rahman M, Sabir AS, Rahman MZ, Islam MT (2017). Chitosan and plant probiotics application enhance growth and yield of strawberry. Biocatal. Agric. Biotechnol..

[95] Desai KG (2016). Chitosan Nanoparticles Prepared by Ionotropic Gelation: An Overview of Recent Advances. Crit. Rev. Ther. Drug Carrier Syst..

[96] Phuong NT (2015). Enzyme-mediated fabrication of an oxidized chitosan hydrogel as a tissue sealant. J. Bioact. Compat. Polym..

[97] Wanichpongpan, P., Suriyachan, K. & Chandrkrachang, S. Effects of chitosan on the growth of *Gerbera* flower plant (*Gerbera jamesonii*), in *Chitin and Chitosan in Life Science* (ed. Uragami, T., Kurita, K. & Fukamizo, T.) 198–201 (Kodansha Scientific, 2001).

[98] Anusha, J. R. & Fleming, A. T. Synthesis and characterization of chitosan nano-aggregates from gladius of *Uroteuthis duvauceli. Int. J. Biomater*. Article ID 5379424. 10.1155/2016/5379424 (2016). 10.1155/2016/5379424 PMC4764718 26977152

[99] Solanki PR, Patel MK, Ali A, Malhotra BD (2015). Chitosan modified nickel oxide platform for biosensing applications. J. Mater. Chem. B..

[100] Vijayalakshmi K, Devi BM, Sudha PN (2016). Synthesis, characterization and applications of nanochitosan/sodium alginate/microcrystalline cellulose film. J. Nanomed. Nanotechnol..

[101] Ghadi A, Mahjoub S, Tabandeh F, Talebnia F (2014). Synthesis and optimization of chitosan nanoparticles: Potential applications in nanomedicine and biomedical engineering. Casp. J. Intern. Med..

[102] Elamawi RM, Al-Harbi RE, Awatif A, Hendi AA (2018). Biosynthesis and characterization of silver nanoparticles using *Trichoderma longibrachiatum* and their effect on phytopathogenic fungi. Egypt. J. Biol. Pest. Co..

[103] Goy RC, De Britto D, Assis OBG (2009). A Review of the antimicrobial activity of chitosan. Polymers.

[104] Kulikov SN (2014). Antifungal activity of oligochitosans (short chain chitosans) against some Candida species and clinical isolates of Candida albicans: Molecular weight-activity relationship. Eur. J. Med. Chem..

[105] Zhai X (2016). Synthesis and characterization of chitosan-zinc composite electrodeposits with enhanced antibacterial properties. RSC Adv..

[106] Wang X, Du Y, Fan L, Liu H, Hu Y (2005). Chitosan- metal complexes as antimicrobial agent: Synthesis, characterization and structure-activity study. Polym. Bull..

[107] Pandian CJ, Palanivel R, Dhanasekaran S (2016). Screening antimicrobial activity of nickel nanoparticles synthesized using *Ocimum sanctum* leaf extract. J. Nanopart..

[108] Winter M, Samuels PL, Hanson LKO, Macky RD, Kinkel LL (2019). Biological control of Fusarium crown and root rot of wheat by *Streptomyces* isolates- Its complicated. Phytobiomes J..

[109] Qostal S (2020). Management of wheat and barley root rot through seed treatment with biopestcides and fungicides. Plant Cell Biotechnol. Mol. Biol..

[110] Sagar SD, Kulkarni S, Hegde YR (2007). Management of rhizome rot of ginger by botanicals. Int. J. Plant Sci..

[111] Manstretta V, Gourdain E, Rossi V (2015). Deposition patterns of *Fusarium graminearum* ascospores and conidia within a wheat canopy. Eur. J. Plant Pathol..

[112] Ahmed AIS (2017). Chitosan and silver nanoparticles as control agents of some Faba Bean Spot diseases. J. Plant Pathol. Microbiol..

[113] Gondal AS, Ijaz M, Riaz K, Khan AR (2012). Effect of different doses of fungicide (Mancozeb) against *Alternaria* Leaf Blight of Tomato. J. Plant Pathol. Microbiol..

[114] Alcaraz AGL (2016). Enhanced Antifungal Effect of Chitosan/Pepper Tree (*Schinus molle*) Essential Oil Bionanocomposites on the Viability of *Aspergillus parasiticus* spores. J. Nanomater..

[115] Subban, K., Subramani, R., Srinivasan, V. P. M., Johnpaul, M. & Chelliah, J. Salicylic acid as an effective elicitor for improved taxol production in endophytic fungus *Pestalotiopsis microspore*. *PLoS ONE* **14**(2), e0212736. 10.1371/journal.pone.0212736 (2019). 10.1371/journal.pone.0212736 PMC6386501 30794656

[116] Dananjaya SHS, Udayangani RMC, Oh C, Nikapitiya C, Lee J, Zoysa MD (2017). Green synthesis, physio-chemical characterization and anti-candidal function of a biocompatible chitosan gold nanocomposite as a promising antifungal therapeutic agent. RSC Adv..

[117] Santori A, Infantino A (2009). Concia*:* ruolo strategic contro il mal del piede del frumento. L’Informatore Agrario..

[118] Orzali L, Forni C, Riccioni L (2014). Effect of chitosan seed treatment as elicitor of resistance to *Fusarium graminearum* in wheat. Seed Sci. Technol..

[119] Guha SK, Sindhu SS (2011). Disease control and plant growth promotion of green gram by siderophore producing *Pseudomonas* sp. Res. J. Microbiol..

[120] Abdul- Baki, A. A. & Anderson, J. D. Seed biology in *Physiological and biochemical deterioration of seeds* (ed. Kozlowski, T. T.) 283–315 (Agris, 1973).

[121] Sen SK, Chouhan D, Das D, Ghosh R, Mandal P (2020). Improvisation of salinity stress response in mung bean through solid matrix priming with normal and nano-sized Chitosan. Int. J. Biol. Macromol..

[122] Mosmann T (1983). Rapid colorimetric assay for cellular growth and survival: application to proliferation and cytotoxicity assays. J. Immunol. Methods.

[123] Denizot F, Lang R (1986). Rapid colorimetric assay for cell growth and survival, modifications to the tetrazolium dye procedure giving improved sensitivity and reliability. J. Immunol. Methods.

